# Potential for Genetic Improvement of Sugarcane as a Source of Biomass for Biofuels

**DOI:** 10.3389/fbioe.2015.00182

**Published:** 2015-11-17

**Authors:** Nam V. Hoang, Agnelo Furtado, Frederik C. Botha, Blake A. Simmons, Robert J. Henry

**Affiliations:** ^1^Queensland Alliance for Agriculture and Food Innovation, The University of Queensland, St. Lucia, QLD, Australia; ^2^College of Agriculture and Forestry, Hue University, Hue, Vietnam; ^3^Sugar Research Australia, Indooroopilly, QLD, Australia; ^4^Joint BioEnergy Institute, Emeryville, CA, USA

**Keywords:** sugarcane, biofuels, biomass for biofuels, biofuel traits, association studies

## Abstract

Sugarcane (*Saccharum* spp. hybrids) has great potential as a major feedstock for biofuel production worldwide. It is considered among the best options for producing biofuels today due to an exceptional biomass production capacity, high carbohydrate (sugar + fiber) content, and a favorable energy input/output ratio. To maximize the conversion of sugarcane biomass into biofuels, it is imperative to generate improved sugarcane varieties with better biomass degradability. However, unlike many diploid plants, where genetic tools are well developed, biotechnological improvement is hindered in sugarcane by our current limited understanding of the large and complex genome. Therefore, understanding the genetics of the key biofuel traits in sugarcane and optimization of sugarcane biomass composition will advance efficient conversion of sugarcane biomass into fermentable sugars for biofuel production. The large existing phenotypic variation in *Saccharum* germplasm and the availability of the current genomics technologies will allow biofuel traits to be characterized, the genetic basis of critical differences in biomass composition to be determined, and targets for improvement of sugarcane for biofuels to be established. Emerging options for genetic improvement of sugarcane for the use as a bioenergy crop are reviewed. This will better define the targets for potential genetic manipulation of sugarcane biomass composition for biofuels.

## Introduction

Plant biomass from grasses such as sugarcane or woody species contains mostly cellulose, hemicellulose, and lignin (also referred to as lignocellulosic biomass), which can be converted to biofuels as a source of renewable energy. At the moment, plant biomass-derived biofuels have great potential in countries that have limited oil resources because they reduce the dependence on fossil fuel, mitigate air pollution by cutting down greenhouse gas emissions, and can be produced from a wide range of abundant sources (Matsuoka et al., 2009). Biofuels generated from plant lignocellulosic biomass (also known as the second generation of biofuels) have been shown to be advantageous over the first generation (from plant starches, sugar, and oil) in terms of net energy and CO_2_ balance and, more importantly, they do not compete with food industries for supplies (Yuan et al., 2008). To date, producing bioethanol from the sugar in sugarcane has been one of the world’s most commercially successful biofuel production systems, with the potential to deliver second-generation fuels with a high positive energy balance and at a relatively low production cost (Yuan et al., 2008; Botha, 2009; Matsuoka et al., 2009). The rapid growth and high yield of sugarcane compared to other grasses and woody plants makes it a good candidate for ethanol processing platform and the second generation of biofuels in general (Pandey et al., 2000). Sugarcane has an exceptional ability to produce biomass as a C4 plant with the potential of a perennial grass crop allowing harvest four to five times by using ratoons without requiring replanting (Verheye, 2010), resulting in a lower cost of energy production from sugarcane than for most of the other potential sources of biomass (Botha, 2009). Brazil is the world’s first country to launch a national fuel alcohol program (ProAlcooL). This program is based on the use of sugarcane and substitutes the usage of gasoline by ethanol (Dias De Oliveira et al., 2005). Approximately, 23.4 billion liters (6.19 billion U.S. liquid gallons) of ethanol was produced in Brazil in the year 2014 (Renewable Fuels Association, 2015). As of 2009, sugarcane bagasse contributes to about 15% of the total electricity consumed in Brazil, and it is predicted that energy generated from sugarcane stalks could supply more than 30% of the country energy needs by 2020 and will be equal to or more than the electricity produced from hydropower (Matsuoka et al., 2009).

Conventionally, sugarcane bagasse is usually burned to produce fertilizer or steam and electricity to fuel the boilers in sugar mills (Pandey et al., 2000). Recently, it has been used for biofuel production; however, the production cost of biofuels from lignocellulosic biomass is still considered to be relatively high, which makes it difficult to be price-competitive and commercialized on a large scale (Halling and Simms-Borre, 2008). At the moment, the cost of bagasse pretreatment (to remove or separate its recalcitrant components before converting to biofuels) and microbial enzymes contributes mostly to the total production cost, resulting in reducing the incentive to transition from first generation to second generation of biofuels in sugarcane (Yuan et al., 2008). To maximize the efficiency of conversion of sugarcane biomass into biofuels, it is imperative to generate improved sugarcane cultivars with not only high biomass yield and fiber content but also better biomass degradability for conversion to biofuels in addition to improving the pretreatment and enzyme digestion technologies.

This review focuses on the potential for the genetic improvement of sugarcane as a source of biomass for biofuels, exploring the beneficial characteristics of sugarcane, the available genetic resources and germplasm, the potential of cell wall modification by breeding and biotechnology, and the potential of whole genome/transcriptome sequencing applications in dissecting important biofuel traits to improve sugarcane biomass composition. This will define the targets for potential genetic manipulation and better exploitation of sugarcane biomass for biofuels.

## Sugarcane at a Quick Glance

### Biology and Genetics

Taxonomically, sugarcane belongs to the genus *Saccharum* (established by Carl Linnaeus in 1753), in the grass family *Poaceae* (or *Gramineae*), subfamily *Panicoideae*, tribe *Andropogoneae*, subtribe *Sacharinae*, under the group *Saccharastrae* and has a very close genetic relationship to sorghum and other grass family members such as *Erianthus* and *Miscanthus* (Amalraj and Balasundaram, 2006). Typically, the genus is divided into six different species namely *Saccharum barberi, Saccharum edule, Saccharum officinarum, Saccharum robustum, Saccharum sinense*, and *Saccharum spontaneum* (Daniels and Roach, 1987; Amalraj and Balasundaram, 2006), in which *S. spontaneum* and *S. robustum* are wild species; *S. officinarum*, *S. barberi*, and *S. sinense* are early cultivars while *S. edule* is a marginal specialty cultivar. All genotypes of the *Saccharum* genus are reported to be polyploid with the ploidy level ranging from 5× to 16× and are considered as among the most complex plant genomes (Manners et al., 2004). The cytotype (2*n*, the number of chromosomes in the cell) was reported to be different in each species as follows: *S. officinarum* (2*n* = 80), *S. spontaneum* (2*n* = 40–128), *S. barberi* (2*n* = 111–120), *S. sinense* (2*n* = 81–124), *S. edule* (2*n* = 60–80), and *S. robustum* (2*n* = 60, 80); hence, the basic chromosome number (x, the monoploid set of chromosomes in the cell) ranges from 5, 6, 8, 10 to 12 (Sreenivasan et al., 1987). The basic chromosome number of *S. spontaneum* is 8 (even though a number of very variable cytotype is observed) and of *S. officinarum* and *S. robustum* is 10 [Panje and Babu, 1960, D’Hont et al. (1998), and Piperidis et al. (2010)]. For the other three species, *S. sinense, S. barberi*, and *S. edule*, due to the fact that these are early interspecific hybrid cultivars, there have not been a consensus reported, but a study by Ming et al. (1998) suggested that the basic chromosome number for these three species could also be 10.

Hybrid sugarcane was derived from crosses between a female *S. officinarum* (2*n* = 80) and a male *S. spontaneum* (2*n* = 40–128). Due to the female restitution phenomenon, at first, the F1 hybrid conserves the whole *S. officinarum* chromosome set and half of the *S. spontaneum* which was 2*n* + *n*, then a few backcrosses later, this hybrid breaks down to *n* + *n*, establishing the hybrid chromosome set of modern sugarcane hybrid (Bremer, 1961). For this reason, current sugarcane cultivars (*Saccharum* spp. hybrids) have a combination of a highly aneuploid and interspecific set of chromosomes. By using genomic *in situ* hybridization (GISH) and fluorescent *in situ* hybridization (FISH), it is revealed that among chromosomes in the nucleus of modern hybrid sugarcane, approximately 80% are contributed by *S. officinarum*, 10–20% from *S. spontaneum*, and less than 5–17% from recombination of chromosomes of the two species (D’Hont et al., 1996; Piperidis et al., 2001; Cuadrado et al., 2004). Modern sugarcane hybrids are normally crosses between varieties/clones, which makes the combination of the chromosomes in each offspring unique and unpredictable due to the random sorting of the chromosomes in the genome (Grivet and Arruda, 2002). The first sugarcane breeding program, which started more than one century ago, generated a few interspecific hybrids and constitutes the basic germplasm used by sugarcane breeding programs (Ming et al., 2010). Modern sugarcane cultivars are derived from the basic germplasm, but there has been only a few generations for chromosome recombination opportunities (the number of meiosis that chromosomes have undergone is mainly about 2–7) as the sugarcane breeding processes normally take between 10 and 15 years (Raboin et al., 2008; Ming et al., 2010). As a result, the modern sugarcane population has a narrow genetic basis and high linkage disequilibrium (Roach, 1989; Lima et al., 2002; Raboin et al., 2008).

### The Nature of a Complex, Polyploid, and Repetitive Genome

The complex and polyploid genome of sugarcane makes the process of analyzing and understanding difficult by normal methods applied to diploid plants. The size of the sugarcane genome is about 10 Gb while its genome complexity is due to the mixture of euploid and aneuploid chromosome sets with homologous genes present in from 8 to 12 copies (Souza et al., 2011). The estimated monoploid genome size is about 750–930 Mb (the monoploid genome size of the two parental species, *S. officinarum* and *S. spontaneum*, are 930 Mb and 750 Mb, respectively), not much larger than the sorghum genome (~730 Mb) and about twice the size of the rice genome (~380 Mb) (D’Hont and Glaszmann, 2001). On the other hand, studies revealed that despite this complex and polyploid genome, sugarcane showed synteny with other grasses, especially sorghum (collinear, due to the limited divergence time) and maize (orthologous but altered loci collinearity) [reviewed in Grivet and Arruda (2002)]. It was thought that the sugarcane genome contains roughly the same amount of repetitive DNA as in the sorghum genome (Jannoo et al., 2007); however, studies on BAC-end sequences by Wang et al. (2010), Figueira et al. (2012), and Kim et al. (2013) suggested that there is less repetitive content in the sugarcane genome (e.g., 45.2% and 42.8% repetitive sequences observed in large BAC collections in comparison to 61% in the sorghum genome). More recently, using the *k*-mer approach, Berkman et al. (2014) found that the repetitive proportion in three sugarcane hybrid cultivars ranges from 63.74 to 78.37% and higher than that in the sorghum genome (55.5%) using the same approach. The authors postulated that the increased proportion could be attributed to ploidy level rather than repetitive content in the sugarcane genome. A high gene-copy number, the integration of two chromosome sets from two different species, and a significant repeat content hinder the understanding of how the genome functions and obtaining a genuine assembled monoploid genome (Souza et al., 2011; Figueira et al., 2012).

### Candidate Crop for Future Biofuels

To date, sugarcane is among the most efficient crops in the world together with other C4 grasses such as switch grass (*Panicum virgatum*), *Miscanthus* species (*Miscanthus x giganteus*), and *Erianthus* species (*Erianthus arundinaceus* Retz.) in terms of converting solar energy into stored chemical energy and biomass accumulation (Tew and Cobill, 2008; Furtado et al., 2014). In general, C4 plants outperform C3 plants in biomass yield, including grain, stem, and leaf yield (Jakob et al., 2009; Wang and Paterson, 2013). Sugarcane and other C4 grasses are the highest yield potential feedstocks (Table 1), and for sugarcane, the potential yield can exceed 100 tons dry matter per hectare per year (Jakob et al., 2009; Moore, 2009; Henry, 2010a). At present, the most suitable energy crop is probably sugarcane because of its high biomass yield and the potential for production on other than prime agricultural land avoiding competing with the land used for food industries (Waclawovsky et al., 2010). Globally, sugarcane is the most important crop in about 100 countries with a production area of 26.9 million hectares, total production of ~1.9 billion tons, and yield of 70.9 tons of fresh cane per hectare (FAOSTAT, 2015). At present, Brazil is the world’s largest sugarcane producer followed by India, China, Thailand, Pakistan, Mexico, Colombia, Indonesia, Philippines, U.S., and Australia (FAOSTAT, 2015). In sugarcane internodal tissue, sucrose concentration ranges from 14 to 42% of the dry weight (Whittaker and Botha, 1997), while the rest of dry biomass comes from the cell wall lignocellulose, mostly containing cellulose, hemicellulose, lignin, and ash (Pereira et al., 2015). Biofuels from sugarcane can be produced extensively not only from its soluble sugar but also from main residues in sugarcane production, bagasse and trash, on the same production area (Seabra et al., 2010; Alonso Pippo et al., 2011a,b; Macrelli et al., 2012). The total estimated available lignocellulosic biomass from sugarcane worldwide was 584 million dry tons per year, with an average lignocellulosic biomass yield of 22.9 dry tons per hectare per year (Van Der Weijde et al., 2013). Sugarcane bioethanol yield from bagasse is estimated at about 3,000 L per hectare in a total yield of 9,950 L per hectare from sugar and bagasse (Somerville et al., 2010).

**Table 1:** **Average lignocellulosic biomass yield (dry matter) from sugarcane compared to other sources**.

Plant name	Yield (tons/ha/year)	Reference
Sugarcane	22.9^a^	Van Der Weijde et al. (2013)
Switch grass	7–35	Reviewed in Hattori and Morita (2010)
Miscanthus	12–40	Reviewed in Hattori and Morita (2010)
Erianthus	40–60	Reviewed in Hattori and Morita (2010)
Eucalyptus	15–40	Reviewed in Johansson and Burnham (1993)

*^a^Average total cane biomass dry matter is 39 tons/ha/year (Moore, 2009)*.

## Available Sugarcane Genetic Resources for Biofuels

### Existing Variations within *Saccharum* Germplasm

Genetically diverse sugarcane germplasm may play a key role in improving sugarcane for biofuels through breeding and biotechnological approaches. Genetic variation may be found in biomass yield, fiber content, and sugar composition in the *Saccharum* germplasm. This includes the diversity among the cultivars within one species and also diversity among species within the genus. A relatively high genetic variability within sugarcane hybrid cultivars was reported thanks to their heterozygosity and high polyploidy despite their originating from a few clones of a narrow genetic base (Aitken and McNeil, 2010). There is also great genetic and morphological diversity within *Saccharum* species, *Miscanthus* species, and *Erianthus* species to be potentially exploited and incorporated to broaden the genetic base in breeding programs (Harvey et al., 1994; Aitken and McNeil, 2010). To date, the genetic diversity of *S. officinarum* has been exploited in breeding programs; however, the diversity of *S. spontaneum* and other species have not been used much (Aitken and McNeil, 2010). *Saccharum* species have also been shown to have varied genome size, *S. officinarum* genome is about 7.50–8.55 Gb, *S. robustum* ranging from 7.56 to 11.78 Gb, and *S. spontaneum* ranging from 3.36 to 12.64 Gb, whereas the other three species – *S. sinense, S. barberi, S. edule* – and modern sugarcane are interspecific hybrids whose genome size depends upon each cross (Zhang et al., 2012).

There are two world largest collections of germplasm of *Saccharum* species, one is located in Florida (USA) while the other is in Kerala (India), containing approximately 1,200 accessions collected from 45 countries (Tai and Miller, 2001; Todd et al., 2014). These collections could be potentially selected and utilized for breeding purpose to improve sugarcane germplasm for new biofuel traits (Todd et al., 2014). The wild sugarcane species show wider variability in comparison to the domesticated species. In the *Saccharum* genus, *S. spontaneum* has the widest range of morphological variability, ratoon yielding, as well as biotic and abiotic stress tolerance (Tai and Miller, 2001; Aitken and McNeil, 2010; Govindaraj et al., 2014). The coefficient of variation (CV%) for some of the traits such as internode length, midrib width, leaf width, plant height, and stalk height studied by Govindaraj et al. (2014) were reported to be between 15 and 30%, which indicates a very high variability within the collection. It has been shown that the diversity within modern sugarcane hybrids was mostly contributed by the introgression from *S. spontaneum* (D’Hont et al., 1996). On the other hand, *S. robustum* also possesses a large amount of phenotypic variations in many traits studied (Aitken and McNeil, 2010). Sugarcane parental species (*S. officinarum*, *S. spontaneum*, and *S. robustum*), *Miscanthus* species, *Erianthus* species, and sorghum species with their diversity in genome content, structure, and tremendous allelic variation are a valuable and significant genetic reservoir which could be exploited for improving sugarcane biomass.

### Genetic Markers and Maps

To support the effort of understanding the sugarcane genome, many physical maps, molecular markers, and resources such as RFLP, RAPD, AFLP, SSR, and ESTs have been developed over time. These common markers have been applied for genetic studies such as diversity, mapping, quantitative trait loci (QTL), and synteny definition; however, these systems have been developed mostly for well-established diploid species and are less effective for polyploidy plants (Garcia et al., 2013). Markers like AFLP, SSR, and RFLP are unable to estimate the number of allelic copies and level of polyploidy in complicated genomes such as potato, strawberry, and sugarcane (Garcia et al., 2013). More recently, the use of SNPs markers, which are distributed at high density across the genome, for complex genomes can allow estimation of the number of allelic copies and the ploidy level of genomes (Zhu et al., 2008; Hall et al., 2010). The currently available genetic maps and markers have been generated for sugarcane by using low-throughput methods, providing limited information on genome organization due to the low density of markers and coverage (most of them have less than 1,000 markers) (Aitken et al., 2014). Therefore, it is difficult to allocate these markers into linkage groups or cosegregation groups or sugarcane expected chromosome number (Souza et al., 2011). More detailed linkage maps of *S. officinarum* cultivar IJ76-545 (534 markers in 123 linkage groups) and cultivar Green German (615 markers in 72 linkage groups); *S. spontaneum* cultivar IND (536 markers in 69 linkage groups); and the hybrid cultivars R570 and Q165 (with 2,000 markers placed in more than 100 linkage groups) have been constructed (Souza et al., 2011; Aitken et al., 2014). Most recently, using Diversity Array Technology (DArT), Aitken et al. (2014) integrated DArT markers, RFLPs, AFLPs, SSRs, and SNPs into the largest marker collection for sugarcane, which contains 2,467 single-dose markers for the cross between Q165 and IJ76-514 (a *S. officinarum* accession) and 2,267 markers from the cultivar Q165. These were placed into 160 linkage groups and eight homology groups, with some uncategorized linkage groups indicating that more markers are required. There is still a need to develop high-throughput marker arrays for sugarcane association studies, to generate more markers, and also to make use of the available markers. These markers will be a valuable resource in facilitating and unraveling the complex genome structure of sugarcane. It is worth considering that information on DNA-based molecular markers of progenitor plants can potentially reveal available genetic polymorphism for the analysis of their progenies (Henry et al., 2012). This could be a useful strategy in the case of sugarcane, where the genomes of the parental species are less complex than that of the hybrids.

### Transcriptome Sequences and Transcription Factors

Expressed sequence tags (ESTs) and complementary DNA (cDNA) sequences provide direct evidence of the genes present in the samples, and this sequence information is very useful for genome exploration, gene prediction/discovery, genome structure identification, SNP characterization, and transcriptome and proteome analysis (Nagaraj et al., 2007). As of May 2015, the GenBank EST database (dbEST) was composed of 75,906,308 ESTs from different organisms of which 284,818 hits were detected under the search term sugarcane (“*S. officinarum*” or “*Saccharum* hybrid cultivar” or sugarcane). In the last 20 years, sugarcane ESTs have been used for gene discovery, BAC clone selection, and dissecting the coding regions of the genome, involving many projects in South Africa, France, U.S., Australia, and Brazil (Carson and Botha, 2000, 2002; Vettore et al., 2001; Casu et al., 2003, 2004; Grivet et al., 2003; Pinto et al., 2004; Bower et al., 2005). The largest collection of sugarcane ESTs was generated by SUCEST, which is composed of approximate 238,208 ESTs from 26 diverse cDNA libraries of different tissues of sugarcane cultivars, e.g., SP80-3280, SP70-1143, RB845205, RB845298, and RB805028 (Vettore et al., 2001, 2003; Souza et al., 2011). These sequences were assembled into 42,982 sugarcane assembled sequences representing more than 30,000 unique genes (~90% of the estimated genes, about 43,141, of *S. officinarum*) (Vettore et al., 2003; Hotta et al., 2010; Grassius: Grass Regulatory Information Server, 2015). There are other sugarcane EST collections containing less EST entries generated by Casu et al. (2003, 2004) (8,342 ESTs), Ma et al. (2004) (7,993 ESTs), Gupta et al. (2010) (~35,000 ESTs) and small number of ESTs by Carson and Botha (2000, 2002).

Due to the homology between genomes, genome-wide mapping of ESTs of one species provides an important framework for the genome structure of other related species (Sato et al., 2011). However, it is noteworthy that the discovery of the ESTs may be restricted to specific cultivars, as within sugarcane germplasm each cultivar has been shown to have different gene expression level [reviewed in Hotta et al. (2010)]. Moreover, for biofuel trait analysis, the TFs regulating monolignol biosynthesis in lignin pathway have received attention as understanding this allows reducing and modifying lignin content and composition which are essential in addressing the recalcitrant problem in biomass conversion (Santos Brito et al., 2015). It is shown that the lignin regulation can be species specific and information on TFs obtained from model plants such as *Arabidopsis* may require to be validated in other species (Santos Brito et al., 2015). A limited number of TFs in grass and sugarcane have been preliminarily characterized recently including those involved in monolignol biosynthesis, for example, in grass (Handakumbura and Hazen, 2012), rice (Yoshida et al., 2013), sorghum (Yan et al., 2013), and sugarcane (Santos Brito et al., 2015). Gene discovery of sugarcane has progressed to some extent despite the complexity of the genome. The valuable information of ESTs, TFs, full-length cDNAs, and BACs provides an understanding of allelic variations in the genome while a full-genome sequence is not available.

### BAC Libraries to Construct a Reference Genome for Sugarcane

Sugarcane cultivar R570 and other cultivars including ones from the parental species *S. officinarum* and *S. spontaneum* have been used for constructing of bacterial artificial chromosome (BAC) libraries (Hotta et al., 2010). BAC libraries from the sugarcane include hybrid cultivar R570 (103,296 clones, average insert size of 130 kb and two other libraries of 100,000 clones) (Tomkins et al., 1999; Grivet and Arruda, 2002), *S. spontaneum* cultivar SES208 (38,400 clones, average insert size of 120 kb), and *S. officinarum* cultivar LA Purple (74,880 clones, average insert size of 150 kb) generated from different restriction enzymes, e.g., *Hin*dIII and *Bam*H1 [reviewed in Souza et al. (2011)]. BAC sequencing in sugarcane is currently based on the sequencing of BAC clones anchored to an available physical map. Even though it requires a higher cost compared to the whole-genome shotgun sequencing (using high-throughput platforms, Illumina, for example), it is a reliable approach for reference construction, especially, for highly repetitive genomes which cannot be sequenced and resolved by a short-read method (Eversole et al., 2009; Steuernagel et al., 2009). This BAC sequencing approach has been used successfully in sequencing of *Arabidopsis*, rice, and maize genomes and producing the barley reference genome [reviewed in Steuernagel et al. (2009)]. The ongoing Sugarcane Genome Sequencing Initiative (SUGESI) has selected 5,000 BAC clones for sequencing from a library by Tomkins et al. (1999) of cultivar R570, the most intensively characterized cultivar to date, to help assembly of the monoploid coverage (monoploid tiling path) of the sugarcane genome using the sorghum sequence as the guide (Souza et al., 2011; Sugesi, 2015).

### *Sorghum bicolor* Genome as the Closest Related Reference Genome

Sorghum is the most closely related species to sugarcane (Grivet et al., 1994; Dillon et al., 2007). The sorghum genome sequencing project was initiated and completed in 2007 with the total genome size of ~730 Mb, and 34,496 protein-coding loci, at the coverage of 8.5× using whole-genome shotgun sequencing by standard Sanger methodologies (Paterson et al., 2009). The sequenced genome is composed of 10 pairs of chromosomes and 3,294 supercontigs (most of these have been placed into chunks on 10 chromosomes), covering 90% of the genome and 99% of protein-coding regions (including the majority of available non-repetitive markers, known sorghum protein-coding genes, and the majority of ESTs) (Paterson et al., 2009). The sorghum genome has approximately 61% repetitive DNA, a low level of gene duplication compared to other C4 grasses, and a high degree of gene parallelism with sugarcane, even though the sugarcane genome is much more polyploid (Paterson et al., 2009, 2010). Microcollinearity between sugarcane and sorghum genomes indicated that sorghum is suitable as the template for sugarcane genome assembly (Ming et al., 1998; Wang et al., 2010; Figueira et al., 2012). It has been suggested that the sugarcane genome could be 20–30% smaller than that of sorghum despite the estimated monoploid genome size of sugarcane being about 760–930 Mb, at approximately the size of the sorghum genome (Figueira et al., 2012).

## Biomass-Derived Biofuels and the Challenging Issues in Biomass Conversion to Biofuels

### The Second Generation of Biofuels – Cell Walls for Fuels

Due to the depletion of fossil fuel sources, the potential for oil to become more expensive, and the raising awareness of the negative impact of fossil fuels on the environment, biomass-derived biofuels have been investigated and developed recently as an alternative source of renewable, sufficient, and clean energy (Botha, 2009). The demand for renewable biofuels is predicted to be increasing (Fedenko et al., 2013). The first generation of biofuels from plant biomass involved the process of conversion of stored polysaccharides, non-structural carbohydrates, and oils from plants (starchy, sugary, and oily parts of plants such as corn starch, sugarcane molasses, soybeans, canola seeds, and palm oil) into fuels like ethanol and diesel (Schubert, 2006; Yuan et al., 2008). However, these sources are also used as food supplies and are limited due to the increasing demand from the growing world’s population (Schubert, 2006). The second generation of biofuels can be generated by using the non-food parts of plants such as cell walls, composed of structural polysaccharides, such as cellulose and hemicellulose (Schubert, 2006; Yuan et al., 2008; Henry, 2010a). This is considered to be advantageous over the first generation of biofuels as it has a higher energy production potential, lower cost, sustainable CO_2_ balance, no competition with the food production, and a wide range of plant biomass sources are available at costs affordable to a biorefinery (Yuan et al., 2008; Henry, 2010a). As of 2009, sugarcane biomass as sucrose accounted for about 40% of biofuels feedstock worldwide for first-generation biofuel production (Lam et al., 2009). Using sugarcane bagasse as a feedstock for second-generation biofuels would lead to doubling the current output of biofuel production from sugarcane (Halling and Simms-Borre, 2008).

### Sugarcane Cell Wall and Biomass Composition

Physically, sugarcane biomass can be divided into four major fractions, whose content depends on the industrial process: fiber (heterogeneous organic solid fraction), non-soluble solids (inorganic substances), soluble solids (sucrose, waxes, and other chemicals), and water (Canilha et al., 2012; Shi et al., 2013). Second generation of biofuels focuses on using the fiber fraction especially the cell wall constituents of the plant to produce biofuels (Schubert, 2006; Henry, 2010a). This approach may be made more efficient by optimizing the composition of the biomass source for biofuel production. This could be achieved by advances in pretreatment methods or biotechnological modification of cell wall synthesis pathways to create a biomass that can be more efficiently processed (Sims et al., 2006; Yuan et al., 2008; Simpson, 2009; Viikari et al., 2012). Three major components make up the fiber fraction of sugarcane, namely, cellulose, hemicellulose (or non-cellulosic polysaccharide components), and lignin. Cellulose constitutes around 50% of the dry weight sugarcane bagasse while hemicellulose and lignin each account for about 25% (Loureiro et al., 2011). These three components are biosynthesized through different complex pathways (Higuchi, 1981; Whetten and Ron, 1995; Saxena and Brown, 2000; Mutwil et al., 2008; Harris and DeBolt, 2010; Pauly et al., 2013). Cellulose and hemicellulose molecules form the cell walls which act as the skeleton of plants and are strengthened by lignin and phenolic cross-linkages (Carpita, 1996; Henry, 2010b). The complex interlinking between cell wall components plays an important role in grass defense and yet challenges the biofuel production by requiring the pretreatment to separate them (De O. Buanafina, 2009).

The sugarcane and grass cell wall are categorized as type II cell wall, which differs from the type I and type III cell walls of other plants [reviewed in Souza et al. (2013)]. In general, there is little pectin, less lignin, and less structural proteins in grass cell walls than that in the non-grasses (Carpita, 1996; Henry, 2010b; Saathoff et al., 2011). There is similar cellulose content between grass and non-grass primary and secondary cell walls; however, hemicellulose composition is different between two groups. Grass cell walls have four to eight times more xylans, higher mixed linkage glucans, and lower levels of xyloglucans, mannans, glucomannans, and pectin in primary cell wall, but higher phenolics and lignin in the secondary cell wall (Loureiro et al., 2011). Grassy lignin is composed of three monolignols (lignin syringyl – S, lignin guaiacyl – G and lignin hydroxyphenyl – H subunits) forming various ratios of them and normally has more H subunit (more coumaryl derivatives) than in non-grasses (Vogel, 2008). A recent study by Bottcher et al. (2013) showed that sugarcane lignin content and composition are varied depending on tissue types and stem positions on the plant. Within one plant, the bottom internode has higher lignin accumulation than the top internode, and the inner part of stem has higher syringyl/guaiacyl (S/G) ratio than the outer part. Polysaccharides found in sugarcane leaf and culm walls were similar but different in the proportions of xyloglucan and arabinoxylan (Souza et al., 2013). The major monosaccharides released from sugarcane cell walls were glucose, xylose, and arabinose (Loureiro et al., 2011; Rabemanolontsoa and Saka, 2013; Souza et al., 2013). Understanding the fine structure and detailed composition of sugarcane cell wall will assist in optimizing the tissue pretreatment and cell wall hydrolysis protocol. At present, converting sugarcane lignocellulosic biomass to ethanol includes (1) pretreatment to remove the lignin and other recalcitrant cellular constituents (or hemicellulose) to free cellulose, (2) enzyme-mediated action to depolymerize carbohydrates to simple sugars, and (3) fermentation of sugars and distillation of ethanol as the end product (Canilha et al., 2012).

### Dealing with the Conversion Issues

Even though sugarcane biomass is less resistant to enzymatic digestion compared to that from woody plants, it is reported that biomass recalcitrant components impede the efficiency of the conversion to ethanol (Jung, 1989; Anterola and Lewis, 2002; Chen and Dixon, 2007; Himmel et al., 2007; Balat et al., 2008; Li et al., 2013). Biomass recalcitrance is caused by many factors such as the presence of epidermal and sclerenchyma tissues, vascular bundle density and arrangement, degree of lignification, heterogeneity and complexity of cell wall constituents, insoluble matter, natural inhibitors, and cellulose crystallinity (Himmel et al., 2007). Most approaches for producing biofuels from biomass at the moment rely on the disruption of the biomass, to separate lignocellulose and remove lignin in the biomass, and then conversion using microbial enzymes (Sticklen, 2006). In general, overcoming the recalcitrant issue can be addressed by physical, chemical, and genetic approaches. Physical and chemical strategies deal mainly with the pretreatment and involve loosening the cell wall structure, lowering the biomass heterogeneity, providing the enzymes access to the cellulose, cleaving the crossing linkages, and removing enzymatic inhibitors (Balat et al., 2008; Saathoff et al., 2011). To make the physical and chemical changes in plant biomass, pretreatment processing conditions must be tailored to the specific chemical and structural composition of the various and variable sources of lignocellulosic biomass (Mosier et al., 2005). Currently available physical and chemical pretreatment methods are varied and can be listed as uncatalyzed steam explosion, flow-through acid, liquid hot water, pH-controlled hot water, dilute acid, ammonia, lime and, more recently, the method using ionic liquids (Mosier et al., 2005; Shi et al., 2013; Sun et al., 2013). Genetic approaches involve genetic enhancement, molecular biology, and plant breeding efforts to improve biomass sources by having crops with less lignin, modified lignin, crops that self-produced enzymes, and crops with increased cellulose and biomass overall [reviewed in Sticklen (2006)]. The costs of the enzymatic pretreatment of cellulosic biomass (which accounts for about 25% of total processing expenses), biomass conversion, and microbial tanks limit the price-competitiveness of biofuel from lignocellulosic biomass in comparison to fossil fuel (Gnansounou and Dauriat, 2010; Macrelli et al., 2012, 2014; Van Der Weijde et al., 2013). This emphasizes the value of genetic improvement of biomass composition to reduce processing costs.

## Potential Improvement of Sugarcane by Breeding for Biofuels

The complex and highly polyploid genome of sugarcane poses a great challenge in unraveling and studying its functions. Each cross of modern sugarcane cultivar has a unique set of chromosomes due to the random sorting of chromosomes and recombination of alleles from two progenitor species (Grivet and Arruda, 2002). There are several distinct alleles at each locus in sugarcane chromosomes, making the characteristics of the offspring unpredictable and requiring evaluation of thousands of lines from many parents to gather sufficient information in breeding programs (Matsuoka et al., 2009). In conventional breeding, after crossing and obtaining the F1 generation, hundreds of thousands of F1 seedlings are used for screening for the desired traits such as disease resistance, sugar content, agronomic characteristics, and adaptability (Matsuoka et al., 2009). The process is normally repeated for some vegetatively propagated generations to obtain the required stability of the traits. For industrial purpose, after a long process of selection, from hundreds of thousands seedlings at the beginning, breeders normally end up at a limited number of clones for release as commercial lines or cultivars.

To facilitate the second generation of biofuels, sugarcane breeding programs need to be focusing not only on important traits such as total biomass yield, sugar yield adaptability to local environment, and resistance to major pathogens but also on biofuel traits (e.g., less lignin, improve biomass composition for conversion) as a whole (Matsuoka et al., 2009; Waclawovsky et al., 2010). In sugarcane breeding, to maximize heterosis, the parents are usually selected from divergent genotypes of genetic background (Tabasum et al., 2010). Increasing sugarcane biomass yield and productivity is getting more and more difficult to achieve by conventional methods; hence, broadening the sugarcane genetic basis by introgression of its ancestors or closely related species such as *Miscanthus* and *Erianthus* is being explored in sugarcane improvement [reviewed in Dal-Bianco et al. (2012) and De Siqueira Ferreira et al. (2013)]. This is normally done by crossing *S. officinarum* and *Erianthus*, *Miscanthus*, or backcrossing the hybrids to *S. spontaneum* (Matsuoka et al., 2009). Dual-purpose cane and energy cane, sugarcane lines for lignocellulosic biomass production, have been derived from two sugarcane species, *S. spontaneum* and *S. robustum*, by crossing to develop lines with a high ability to accumulate fiber and high biomass content in addition to accumulating soluble sugars (De Siqueira Ferreira et al., 2013). Another case is *Miscane*, which was the result of crossing between *Saccharum* x *Miscanthus*. This produces cane varieties with more biomass (lignocellulose and total fermentable sugars), disease resistance, and cold tolerance. This effectively adapts *Miscanthus* to a tropical climate and expands sugarcane production to temperate, dry, and cold conditions (Alexander, 1985; Burner et al., 2009; Lam et al., 2009). Recently, using molecular markers in sugarcane breeding program (marker-assisted selection) allows the direct comparison of DNA genetic diversity and provides a precise tool in assessing the genetic diversity of the germplasm (Tabasum et al., 2010; Berkman et al., 2012). The use of markers associated with the desired traits in combination with the advances in next-generation sequencing (NGS) technology, bioinformatics tools, and high-throughput phenotyping methods will significantly improve the sugarcane breeding programs (Lam et al., 2009). NGS will allow a great number of markers such as SNPs to be generated, which could be used to obtain a high density of marker at high coverage across the genome, to dissect the important traits they associate with. These sources of markers will be essential in breeding programs for screening of the parental plants from germplasm collection and of progenies derived from the crosses, selecting traits where the phenotypic methods are not practical (Berkman et al., 2012). High-throughput phenotyping methods will collect data from a large number of samples to overcome the small effects of genes, especially the QTL, controlling the traits (Lam et al., 2009).

## Potential Improvement by Molecular Genetics for Biofuels

The competitiveness of biofuels over other options relies on biotechnology advancement. Efficient conversion of plant biomass to biofuels requires the supply of appropriate feedstocks that can be sustainably produced in large quantities at high yields. The efficient conversion of the biomass in these feedstocks will be facilitated by having a composition that is optimized for efficient processing to deliver high yields of the desired end products. Manipulating of the carbohydrates of the cell walls is the key of improving the biomass composition for biofuels (Harris and DeBolt, 2010). Powerful tools of biotechnology could aim to produce genetically modified sugarcane plants with a favorable ratio of cellulose to non-cellulose content; with *in planta* enzymes that can digest the biomass or degrade the lignin prior to its conversion to ethanol; with pest and disease resistance, flower inhibition, abiotic resistance; or incorporate them into elite sugarcane cultivars for better agronomic performance (Sticklen, 2006; Yuan et al., 2008; Matsuoka et al., 2009; Arruda, 2012).

Among the grasses potentially used for biofuel production such as sugarcane, switch grass, *Miscanthus*, and *Erianthus*, sugarcane has been used more for gene transformation studies (Falco et al., 2000; Manickavasagam et al., 2004; Basnayake et al., 2011) and the first transgenic sugarcane was established by Bower and Birch (1992). The current status of improving sugarcane biomass by using the genetic tools is hindered by its genome complexity, low transformation efficiency, transgene inactivation (gene silencing and regulation), somaclonal variation, and difficulty in backcrossing (Ingelbrecht et al., 1999; Hotta et al., 2010; Arruda, 2012; Dal-Bianco et al., 2012). Targets tackled so far on sugarcane include sucrose and biomass yield increase [i.e., in Ma et al. (2000) and Botha et al. (2001)], downregulation of lignin content or monolignol changes in lignin to lower biomass recalcitrance (described later), expression and accumulation of microbial cellulosic enzymes in leaf [i.e., in Harrison et al. (2011)], herbicide tolerance [i.e., in Gallo-Meagher and Irvine (1996) and Enríquez-Obregón et al. (1998)], disease or pest resistance [i.e., in Joyce et al. (1998), Arencibia et al. (1999), and Zhang et al. (1999)], flowering inhibition [reviewed in Matsuoka et al. (2009) and Hotta et al. (2010)], and drought tolerance [i.e., in Zhang et al. (2006)]. Genetically modified sugarcane has great potential to contribute to biofuel production, with new varieties incorporating these characteristics (Arruda, 2012). Unexploited genes not only from the *Saccharum* germplasm but also in other related species, such as cold-tolerant genes in *S. spontaneum* and *Miscanthus* or drought-tolerant genes in sorghum, once identified would allow their integration into the sugarcane genome, facilitating the production of more sugarcane biomass in temperate areas or under dry conditions (Lam et al., 2009).

Increasing plant cellulose and total biomass content may be achieved by using approaches such as manipulation of growth regulators or key nutrients, increasing the ability of the plant to fix carbon by increasing atmospheric CO_2_ and also manipulating some key metabolic enzymes in biomass synthesis pathways [reviewed in Sticklen (2006)]. Reduction of the cross-links of the maize cell walls (including ferulate and diferulate cross-links; benzyl ether and ester cross-links) has been shown to increase the initial hydrolysis of its cell wall polysaccharides by up to 46% (Grabber, 2005). In general, selection of grasses with less ferulate cross-linking or potent microbial xylanases by breeding or engineering tools are more attractive than pretreatment of the cell wall with a feruloyl esterase (Grabber, 2005).

Lignin content accounts for about 25% of sugarcane total lignocellulosic biomass and is probably the main obstacle affecting the efficiency of saccharification during conversion to ethanol (Canilha et al., 2012, 2013). Lignin and other recalcitrant components in cell walls prevent cellulase accessing the cellulose molecules and need to be removed before further processing (Sticklen, 2006). Lignin biosynthesis pathways are complicated and at least 10 different enzymes have been found involved in the lignin pathway in sugarcane (Higuchi, 1981; Whetten and Ron, 1995) and a total of 28 unigenes associated with monolignol biosynthesis were identified in sugarcane using SUCEST database and annotated genes from closely related species such as sorghum, maize, and rice (Bottcher et al., 2013). Tailoring sugarcane biomass composition for biofuels can be achieved by manipulating some of the key genes in lignin pathway (downregulation of some key enzymes), mostly targeting genes which encode the terminal enzymes such as caffeic acid *O*-methyltransferase (COMT) and cinnamyl alcohol dehydrogenase (CAD), to minimize the impact of the modifications on growth and development of the plant [as reviewed in Sticklen (2006), Jung et al. (2012), and Furtado et al. (2014)]. Not only lignin content but also the lignin S/G ratio is a very important aspect to consider in terms of modifying the lignin content because these two are both associated with biomass recalcitrance (Chen and Dixon, 2007; Li et al., 2010). Sugarcane lignin content was reduced by 3.9–13.7% using RNA interference (RNAi) suppression to downregulate the *COMT* gene [which has at least 31 different ESTs involved (Ramos et al., 2001)] by 67–97% and at the same time, the lignin S/G ratio was reduced from 1.47 to 1.27–0.79 (Jung et al., 2012). This resulted in an increase of up to 29% in total sugar yield without pretreatment (34% with dilute acid pretreatment). This study suggests that RNAi-mediated gene suppression is a promising method for suppression of target genes not only in lignin pathway but also for cell wall constituent biosynthesis (Jung et al., 2012; Bottcher et al., 2013).

Producing enzymes *in planta* is another way to cut the cost of biofuel production as it reduces the expense of enzymes and enzyme treatment. *Cellulase* has been produced within the plant (in the apoplast) of *Arabidopsis*, rice, and maize without effects on the growth and development of the host plants [reviewed in Sticklen (2006)]. *In planta* enzyme expression in sugarcane is still in its infancy; however, a high-yield biofuel plant such as sugarcane must be a target for the production of enzymes within the biomass. Recombinant protein enzymes have been targeted to organelles such as chloroplasts, vacuoles, and the endoplasmic reticulum to separate the enzymes produced and their substrates (Harrison et al., 2011). In sugarcane, thanks to its well-established transformation methods via *Agrobacterium*, the expression of enzymes in leaves and other tissues is feasible (Manickavasagam et al., 2004; Taylor et al., 2008). Endoglucanases and exoglucanases have been overexpressed in sugarcane leaves by using the maize *PepC* promoter achieving an accumulation level of 0.05% of total soluble proteins (endoglucanase, in chloroplast) and less of exoglucanases without altering the phenotype (Harrison et al., 2011). In the future, enzymes might be synthesized in specific energy cane plants that could be coprocessed with other biomass sources from sugarcane for sugar and biomass production (e.g., bagasse from sugar mills) (Arruda, 2012).

## Potential of Sugarcane Whole Genome and Transciptome Sequencing for Biofuels

The advent of NGS technology and a sharp reduction in per-base cost in the past decade [as reviewed in Van Dijk et al. (2014)] allows us to sequence the whole genome of a species, even a complex genome such as sugarcane, at a relatively low price within a relatively short time. At present, the cost of sequencing of a human genome at 30× coverage using the latest Illumina’s Hiseq X is around US $1,000. Since the first plant genome was completely sequenced (*Arabidopsis thaliana* in 2000) using the traditional Sanger sequencing platform, the sequencing strategies have moved to high-throughput and cost-effective approaches (Henry et al., 2012). High-throughput genome sequencing platforms have recently advanced and facilitated improved genotyping, allowing huge data output to be generated for polymorphism detection (especially SNPs) and marker discovery.

### Potential Strategies in Dissection of Biofuel Traits in Sugarcane

At present, a whole-genome sequence of sugarcane is not available to support its biofuel trait analysis. However, a strategy to overcome this using the currently available resources, for dissecting biofuel traits, for example, in sugarcane biomass, is to carry on the association studies, in which a population of genetic variability is selected, phenotyped, and genotyped. Association studies use the molecular markers from the genetic variability to detect the association between markers and traits of interest in order to validate the location of the genes, especially for quantitative traits (Huang et al., 2010). This strategy has been used for human and animal genetic studies since it was first established and more recently also for plants. To date, association studies have been applied successfully to many different plants including *Arabidopsis*, wheat, barley, rice, cotton, maize, potato, soybean, sugar beet, *Pinus*, *Eucalyptus*, ryegrass [also Zhu et al. (2008); for a review, see Hall et al. (2010)], and sugarcane (Aitken et al., 2005; Wei et al., 2006) for important traits like pathogen resistance, flowering time, grain composition, and quality. Association studies differ from traditional QTL studies, where in QTL analysis the linkage disequilibrium between markers and QLTs from a segregating population is established in a cross of different genotypes, whereas in association studies a non-structured population is used (Neale and Savolainen, 2004; Ingvarsson and Street, 2011). Therefore, association studies investigate variations of the whole population not just variations between parents. Association studies analyze the direct linkage disequilibrium between genetic markers and traits to overcome the limitations of the traditional QTL in sample size, low variation, and recombination in the population (Ingvarsson and Street, 2011). In sugarcane, association studies are a powerful method for understanding the complex traits which are controlled by many loci and dosage effects (i.e., Ming et al., 2001; Wei et al., 2006; Banerjee et al., 2015). In general, association studies involve population selection, phenotyping, genotyping, population structure, and statistical testing for the association. For these, there is a requirement to have a population with genetic variability and high linkage disequilibrium; and for sugarcane, the most important aspect of doing association studies is having marker data and a breeding population of elite varieties (Huang et al., 2010). Due to the limited number of generations, low recombination rate between chromosomes, and strong founder effect, it is expected that sugarcane has an extensive linkage disequilibrium despite the large number of chromosomes and being an outcrossing species (Huang et al., 2010). In fact, attaining a F2 population (such as inbred backcrosses or recombinant inbred lines and double haploid lines) in sugarcane is not practical due to its clonal propagation, high heterozygosity, and inbreeding depression (Aitken and McNeil, 2010; Sreedhar and Collins, 2010). Therefore, more commonly, a segregating F1 population from biparental crosses or self-pollinated progenies from heterozygous parents (as the *pseudo* F_2_ population) are used, and hence, most of sugarcane linkage maps (as AFLP, RAPD, isozyme, and SSR) were developed on this type of F1 population (Sreedhar and Collins, 2010). To date, most of these maps have low coverage and a limited number of markers because of the genome complexity and high cost of marker generation (Aitken et al., 2014). The high redundancy of the chromosomes in the sugarcane genome implies that with conventional approaches only the single-dose markers (present on only one of the homologous/homoelogous haplotype) can be used to obtain a high-resolution mapping (Hoarau et al., 2002; Le Cunff et al., 2008).

The potential applications of the current genotyping technologies to sugarcane association studies employ both whole-genome sequencing and whole transcriptome sequencing technologies. Genotyping is normally either by analysis candidate genes or genome-wide approaches, in which the candidate gene approach is restricted to genes which are likely thought to be associated with traits of interest based on prior knowledge (Hirschhorn and Daly, 2005; Ingvarsson and Street, 2011). At present, whole-genome sequencing based on the random sequencing of fragments of whole genomic DNA has been successfully applied to medium-size genomes with limited amount of repetitive elements, genome resequencing with the guide of a reference sequence, or *de novo* assembly of small genomes (Steuernagel et al., 2009; Henry et al., 2012; Xu et al., 2012; Edwards et al., 2013). The large genome size of sugarcane is partially attributable to sugarcane being a polyploid and the genome having a significant amount of repetitive sequences (Berkman et al., 2014). As a result, the current short reads generated from NGS technologies cannot resolve completely the challenges in the sugarcane genomes. For highly repetitive genomes, the genomic complexity will be lost or reduced by using the *de novo* assembly approaches of NGS-derived short reads as the identical repeat sequences in the genome will be collapsed (Green, 2002). Therefore, it is required to develop efficient genotyping strategies using whole-genome sequencing data for sugarcane system to overcome the challenges. Moreover, whole transcriptome sequencing gives details of the entire transcript expressed in the samples across the whole genome and could be applicable to the sugarcane genome in identifying biological significant variations (SNPs) between different developmental stages, between varieties, or for transcripts *de novo* assembly and gene discovery (Henry et al., 2012).

For large and polyploid genomes, there are still requirements to enrich the genomic DNA to capture the coding regions to ensure the depth of coverage, resolve the variable short reads, and lessen the effect of repetitive sequences in the genome on discovery of polymorphisms (Bundock et al., 2012; Henry et al., 2012). Selective sequencing of genomic loci of interest (genes or exomes) can reduce the cost compared to whole-genome sequencing and therefore simplify the data interpretation since non-coding regions are not abundant in the data. The enrichment techniques can be hybrid capture (e.g., Agilent SureSelect, NimbleGen, FlexGen) or selective circularization (e.g., Selector probes) or PCR amplification (e.g., Raindance). Hybrid capture supported by a microarray platform has been applied to sugarcane and other complex genomes due to its high capacity to enrich large regions of interest (1–50 Mb), the possibility of multiplexing, the availability of kits, and a the small amount of input DNA required (<1–3 μg) (Mertes et al., 2011). This approach uses a selection library of fragmented DNA or RNA representing the targets (normally oligonucleotides from 80 to 180 bases produced from known information such as gene indices, ESTs) to capture the cDNA fragments from a shotgun DNA library based on the hybridization and then sequence the captured fragments (Mertes et al., 2011; Bundock et al., 2012). Bundock et al. (2012) conducted the solution-based hybridization (Agilent SureSelect) to capture the exome regions of sugarcane using sorghum and sugarcane coding probes, enriched the genome 10–11 folds, and detected 270,000–280,000 SNPs in each genotype of the material tested. At the moment, a great number of SNPs from a genome or haplotype can be generated by using high-capacity genome sequencing instruments or high-density oligonucleotide arrays (Zhu et al., 2008). The continuous advancement in genotyping technology allows generation of up to 1 million SNPs spanning across the entire genome in one reaction (e.g., using SNP chip), and the newest SNP chip can measure the copy number as well as the allelic variation. Examples of available platforms are Affymetrix (e.g., Affymetrix Genome-Wide Human SNP Array 6.0) and Illumina (e.g., Illumina’s WGGT Infinium BeadChips). Due to the multiple chromosomes in the homologous groups of sugarcane genome and the number of alleles at each locus (and the SNPs numbers consequently), an allele would likely to be defined by a combination of SNPs, not just a single SNP (McIntyre et al., 2006, 2015). SNP genotyping including SNP calling and statistical methods to estimate the ploidy level and allele dosage within homologous groups have been developed for sugarcane by Garcia et al. (2013) to allow in-depth association analysis of the genome. In this study, SNPs were developed by SEQUENOM iPLEX MassARRAY and capture primers and then discovered by QualitySNP software, mass-based procedures, and the SuperMASSA software. For whole transcriptome sequencing, Cardoso-Silva et al. (2014) identified 5,106 SSRs and 708,125 SNPs from the unigenes assembled from RNA-seq data of contrasting sugarcane varieties. These advances in sugarcane genotyping technology, together with well-developed high-throughput phenotyping methods for biofuel traits [reviewed in Lupoi et al. (2013) and Lupoi et al. (2015)] and bioinformatics tools, could accelerate sugarcane analysis while a reference genome is not available.

Some of the association studies have been carried out on sugarcane recently such as those for QTLs which control the *Pachymetra* root rot and brown rust resistance on 154 genotypes (McIntyre et al., 2005); genetics of root rot, leaf scald, Fiji leaf gall, cane sugar, and yield using 1,068 AFLP, 141 SRR (on 154 genotypes), and 1,531 DArT markers (on 480 genotypes) (Wei et al., 2006, 2010); smut and *eldana* stalk borer using 275 RFLP and 1,056 AFLP markers on 77 genotypes (Butterfield, 2007); resistance to sugarcane yellow leaf virus using 3,949 polymorphic markers (DArT and AFLP) on 189 genotypes (Debibakas et al., 2014); markers agro-morphological traits, sugar yield disease resistance, and bagasse content using 3,327 DArT, AFLP, and SSR markers on 183 genotypes (Gouy et al., 2015); and sucrose and yield contributing traits using 989 SSR markers on 108 genotypes (Banerjee et al., 2015). Using the Affymetrix GeneChip Sugarcane Genome Array, Casu et al. (2007) identified 119 transcripts associated with enzymes of cell wall metabolism and development on sugarcane variety Q177. These promising preliminary studies were carried out on small sample sizes and limited numbers of markers (even though a small number of significant associations have been identified) while the polyploid sugarcane genome and small effect of quantitative traits requires larger sample sizes and more markers (e.g., genome-wide markers) so that significant association can be detected (Huang et al., 2010; Gouy et al., 2015).

### The Reference Sequence Matters

As mentioned earlier, construction of a sugarcane nuclear genome reference sequence is an important objective, even though it might take some time to finish. However, in the meantime, sugarcane genome analysis still can exploit the currently available genetic resources such as the sorghum gene indices (sorghum gene models), sugarcane gene indices (DFCI Sugarcane Gene Index version 3.0, an integrated collection of sugarcane ESTs, complete cDNA sequences, non-redundant data of all sugarcane genes and their related information), transcription factors (TFs), and sugarcane tentative consensus/assembled sequences. For example, in the study mentioned earlier, Bundock et al. (2012), based on the gene sequences in the sorghum genome and sugarcane gene indices, captured the exomic regions of two sugarcane genotypes Q165 and IJ76-514, detected SNPs present in 13,000–16,000 targeted genes from Illumina short read data of these samples, and 87–91% of SNPs were validated and confirmed by 454 sequencing. For transcript profiling, the reference transcriptome sequence can be constructed for specific tissues using *de novo* assembly such as in Vargas et al. (2014) and Cardoso-Silva et al. (2014) and validated to find suitable reference gene sets to be used for gene expression normalization as in Guo et al. (2014). The currently available resources, on the other hand, are also utilized. Park et al. (2015) used the Sugarcane Assembled Sequences from SUCEST-FUN database as reference sequences in a study on cold-responsive gene expression profiling of sugarcane hybrids and S*. spontaneum* and found that more than 600 genes are differentially expressed in each genotype after applying stress.

## Conclusion

Sugarcane has been shown to be a good candidate for use as a lignocellulosic biomass feedstock for second-generation biofuel production. However, its genome complexity still remains a great bottleneck restricting the dissection of biofuel traits. The most significant achievements in improving sugarcane biomass for biofuels so far have been the establishment of the high fiber cane varieties to generate more lignocellulosic biomass, and preliminary results in modifying biomass with more cellulose, less lignin content, a preferable lignin S/G ratio, and enzyme expressed *in planta* (in leaves) for easy conversion to biofuels. The improvement of sugarcane biomass has been by traditional breeding, molecular genetics approaches and, more recently, accelerated with the use of NGS technology. The future of second-generation biofuel production using sugarcane lignocellulosic biomass will depend greatly on advances in understanding of the key biofuel traits required to deliver more efficient and price-competitive biofuels. This objective will be facilitated once the whole genome of sugarcane is fully sequenced. Optimizing sugarcane lignocellulosic bagasse composition may result in biomass with better digestibility, modified carbohydrates, and reduction of cross-linking or self-produced enzymes (*in planta*). Currently available sugarcane genetic resources include diverse germplasm in the genus *Saccharum*, genetic markers and maps, ESTs, and the sequence of a closely related species genome. However, novel strategies need to be developed to overcome the challenges posed by the complex genetics. Traditional approaches using breeding and molecular genetics have potential for wider use improving sugarcane while the advent of NGS technology and high-throughput phenotyping technologies will accelerate the process of dissection of biofuel traits, genome-wide. By using these approaches, the loci of interest will be defined for use to improve sugarcane biomass. Once a better understanding of the genes controlling cell wall biosynthesis is achieved, breeding programs will be able to accelerate the selection and development of varieties with optimized biomass composition to generate better sugarcane biomass sources to meet the demand of biofuel production.

## Author Contributions

NVH wrote the paper. AF, FCB, BAS, and RJH discussed and edited the manuscript. All authors read and approved the final manuscript.

## Conflict of Interest Statement

The authors declare that the research was conducted in the absence of any commercial or financial relationships that could be construed as a potential conflict of interest.

## Abbreviations

AFLP, amplified fragment length polymorphism; BAC, bacterial artificial chromosome; CAD, cinnamyl alcohol dehydrogenase (EC 1.1.1.195); cDNA, complementary DNA; COMT, caffeic acid *O*-methyltransferase (EC 2.1.1.68); DArT, diversity array technology; EST, expressed sequence tag; Gb/Mb, gigabase/megabase; LD, linkage disequilibrium; Lignin G, lignin guaiacyl; Lignin H, lignin hydroxyphenyl; Lignin S, lignin syringyl; NGS, next-generation sequencing; QTL, quantitative trait locus; RFLP, restricted fragment length polymorphism; RNAi, RNA interference; S/G ratio, syringyl/guaiacyl ratio; SNP, single nucleotide polymorphism; SUCEST, sugarcane EST database; TF, transcription factor; TIGR, the institute for genome research.
